# A pilot study of bevacizumab combined with etoposide and cisplatin in breast cancer patients with leptomeningeal carcinomatosis

**DOI:** 10.1186/s12885-015-1290-1

**Published:** 2015-04-17

**Authors:** Pei-Fang Wu, Ching-Hung Lin, Ching-Hua Kuo, Wei-Wu Chen, Dah-Cherng Yeh, Hsiao-Wei Liao, Shu-Min Huang, Ann-Lii Cheng, Yen-Shen Lu

**Affiliations:** 1 National Center of Excellence for Clinical Trial and Research, National Taiwan University Hospital, Taipei City, 10002 Taiwan; 2 Department of Oncology, National Taiwan University Hospital, Taipei City, 10002 Taiwan; 3 School of Pharmacy, College of Medicine, National Taiwan University, Taipei City, 10002 Taiwan; 4 Department of Surgery, Taichung Veterans General Hospital, Taichung, 40705 Taiwan; 5 Department of Internal Medicine, National Taiwan University Hospital, Taipei City, 10002 Taiwan; 6 Graduate Institute of Oncology, National Taiwan University College of Medicine, Taipei City, 10002 Taiwan

**Keywords:** Leptomeningeal carcinomatosis, Bevacizumab, Vascular endothelial growth factor (VEGF), Anti-angiogenic therapy, Anti-VEGF therapy

## Abstract

**Background:**

Elevated vascular endothelial growth factor (VEGF) was associated with poor prognosis in leptomeningeal carcinomatosis and anti-angiogenic therapy was found to prolong the survival of mice in preclinical studies. This prospective pilot study investigated the efficacy of anti-VEGF therapy plus chemotherapy in patients with leptomeningeal carcinomatosis originating from breast cancer.

**Methods:**

Eligible patients were scheduled to receive bevacizumab combined with etoposide and cisplatin (BEEP) every 3 weeks for a maximum of 6 cycles or until unacceptable toxicity. The primary objective was the central nervous system (CNS)-specific response rate, which was defined as disappearance of cancer cells in the cerebrospinal fluid (CSF) and an improved or stabilized neurologic status. The impact of VEGF inhibition on etoposide penetration into the CSF was analyzed.

**Results:**

Eight patients were enrolled. The CNS-specific response rate was 60% in 5 evaluable patients. According to intent-to-treat analysis, the median overall survival of the eight patients was 4.7 months (95% confidence interval, CI, 0.3–9.0) and the neurologic progression-free survival was 4.7 months (95% CI 0–10.5). The most common grade 3/4 adverse events were neutropenia (23.1%), leukopenia (23.1%), and hyponatremia (23.1%). The etoposide concentrations in the CSF were much lower than those in plasma, and bevacizumab did not increase etoposide delivery to the CSF.

**Conclusions:**

BEEP exhibited promising efficacy in breast cancer patients with leptomeningeal carcinomatosis. Additional studies are warranted to verify its efficacy and clarify the role of anti-angiogenic therapy in this disease.

**Trial registration:**

ClinicalTrials.gov identifying number NCT01281696.

**Electronic supplementary material:**

The online version of this article (doi:10.1186/s12885-015-1290-1) contains supplementary material, which is available to authorized users.

## Background

Leptomeningeal carcinomatosis results from the spread of cancer cells to the leptomeninges and dissemination within the cerebrospinal fluid (CSF). It has become increasingly common because of the prolonged survival of cancer patients and improvements of diagnostic methods. Approximately 4% − 15% of patients with solid cancers develop leptomeningeal carcinomatosis, and breast cancers, lung cancers, and melanoma are the most common origins. The treatments include intra-CSF and systemic chemotherapy, irradiation, and surgery of bulky metastases. Despite the administration of aggressive treatments, the prognosis is poor, with the median overall survival (OS) ranging from 8 to 16 weeks [1,2].

Recent studies have shown that vascular endothelial growth factor (VEGF) levels in the CSF were significantly higher in patients with leptomeningeal carcinomatosis and correlated with a poor prognosis [3-5]. Reijneveld *et al.* also found that inhibition of angiogenesis prolonged the survival of mice with leptomeningeal carcinomatosis [6]. These findings suggest that VEGF plays pivotal roles in this disease.

Bevacizumab is a recombinant, humanized monoclonal antibody directed against VEGF. It has exhibited efficacy in metastatic breast cancer, colorectal cancer, non-small-cell lung cancer (NSCLC), and glioblastoma multiforme. Our previous study of bevacizumab combined with etoposide and cisplatin (BEEP) demonstrated significant activity for brain metastasis of breast cancer that progressed after whole brain radiation therapy [7]. This pilot study examined the efficacy of BEEP in breast cancer patients with leptomeningeal carcinomatosis. Translational research was performed to evaluate the effects of anti-VEGF therapy on drug delivery to the CSF.

## Methods

### Study design

This prospective, multicenter pilot study was conducted to evaluate the efficacy and safety of BEEP in patients with leptomeningeal carcinomatosis originating from breast cancer. The study was performed at 3 centers in Taiwan from November 2010 to March 2013. The protocol was approved by the research ethics committees of all of the participating centers (National Taiwan University Hospital Research Ethics Committee and Institutional Review Board of Taichung Veterans General Hospital). This trial is registered on ClinicalTrials.gov and has the identification number NCT01281696.

### Eligibility criteria

Patients who had leptomeningeal carcinomatosis originating from breast cancer, based on positive CSF cytology findings, were eligible to participate in this study. Additional inclusion criteria were an age of 18 to 75 years and adequate organ functions and bone marrow reserve.

The major exclusion criteria were prior VEGF-targeted therapy; a history of thrombotic or hemorrhagic disorders; severe nonhealing wounds, ulcers, or bone fractures; regular use of medication that increases bleeding tendency.

Concurrent intrathecal treatment with methotrexate was permitted during the study period. Patients were required to sign an informed consent form before being enrolled in the study.

### Treatment administration

Patients were scheduled to receive BEEP (15 mg/kg of bevacizumab on Day 1; etoposide at 70 mg/m^2^/d from Day 2 to Day 4; and cisplatin at 70 mg/m^2^/d on Day 2) every 3 weeks for a maximum of 6 cycles or until a level of unacceptable toxicity was reached. The use of prophylactic G-CSF (granulocyte colony-stimulating factor) was allowed.

In the first cycle, some modifications of the treatment schedule for the translational research were introduced. Etoposide was administered from Day 1 to Day 3, and bevacizumab was administered 6 hours after etoposide infusion was completed on Day 1.

### Cerebrospinal fluid concentration of etoposide

Patients who had an Ommaya reservoir were subjected to translational research to assess the effects of anti-VEGF treatment on the delivery of etoposide to the CSF. The temporal changes in the etoposide concentration in the CSF and plasma were determined using ultrahigh-performance liquid chromatography with tandem mass spectrometry, as previously described [8].

### Efficacy assessments

Clinical evaluations, including physical, neurological, and CSF cytology examinations, were performed at the baseline and during the study. Tumor-associated neurological signs and symptoms were assessed based on the criteria used by Lin *et al.* [9]. Cytologic negative conversion was defined as the absence of malignant cells in the CSF 2 times in succession. A CNS-specific response was defined as a negative conversion according to the CSF cytology results and a stable or improved neurological status. Patients whose CSF cytology results were persistently positive or positive after only one negative cytology result was obtained were considered nonresponders. Neurologic progression was defined as the observation of positive cytology results after confirmation of a negative conversion, or evidence of leptomeningeal disease progression upon neurological examination [10,11]. All patients were followed until death.

### Safety assessments

Adverse events (AE) were assessed and graded according to NCI CTCAE v3.0 (National Cancer Institute Common Terminology Criteria for Adverse Events). The patients were followed for safety until at least 30 days after discontinuation of the study drug. Severe AEs were defined according to International Conference on Harmonization Good Clinical Practice guidelines. The safety profile was evaluated by recording the incidence and severity of AEs.

### Study objectives

The primary end point of the study was the CNS-specific response rate. Secondary end points included neurologic progression-free survival (PFS) and OS. Furthermore, the study evaluated the impact of VEGF inhibition on etoposide penetration into the CSF.

### Statistics

OS was defined as the time from the initiation of the study medications until death from any cause or the date of last contact with the patient. Neurologic PFS was defined as the time from the initiation of the study medications until the earliest date of neurologic disease progression or death from any cause. OS and PFS estimates were obtained using Kaplan–Meier survival curves. Continuous variables are reported as means and ranges. Categorical variables are reported as frequencies and percentages. All statistical evaluations were performed using SPSS 15.0. A statistical difference was considered to be significant when *P* < .05.

## Results

### Patient characteristics

A total of 8 patients were enrolled in the study, and their baseline clinical characteristics are listed in Table 1. The median age was 55 years (range, 30–65 y) and the median ECOG (Eastern Cooperative Oncology Group) performance status was 2.5 (range, 2–3). Three patients (38%) had received hormone therapy, 2 patients (25%) had received HER2-targeted therapy, and 8 patients (100%) had received chemotherapy for their primary disease; furthermore, 2 patients (25%) had undergone surgery, 4 patients (50%) had undergone radiotherapy, and 6 patients (75%) had received intrathecal chemotherapy for CNS metastases. Five patients (63%) exhibited leptomeningeal metastasis according to MRI examination. Systemic disease outside the central nervous system (CNS) was not under control at the time of leptomeningeal carcinomatosis diagnosis in 5 patients (63%).

**Table 1:** **Patient characteristic at baseline**

	Patients (*N* = 8)
Age, median (range), years	55 (30–65)
Histology, *N* (%)	
Invasive ductal carcinoma	6 (75%)
Invasive lobular carcinoma	1 (13%)
Unknown	1 (13%)
Hormone receptor status, *N* (%)	
ER+ and PR+	3 (38%)
ER- and PR-	5 (63%)
HER2 expression, *N* (%)	
IHC 0-2+ and/or FISH-	3 (38%)
IHC 2+ and FISH+, IHC3+	2 (25%)
Triple negative, *N* (%)	
Yes	3 (38%)
No	5 (63%)
ECOG performance status, *N* (%)	
<2	0 (0%)
2	4 (50%)
3	4 (50%)
Chemotherapy lines in metastatic setting, *N* (%)	
0	1 (13%)
1	2 (25%)
2	2 (25%)
≧3	3 (38%)
Coexisting brain parenchymal metastasis, *N* (%)	
Yes	7 (88%)
No	1 (13%)
Prior therapy for CNS metastasis, *N* (%)	
Surgery	2 (25%)
Radiotherapy	4 (50%)
Numbers of metastatic sites, *N* (%)	
Median (range)	2 (1–4)
1	2 (25%)
2	4 (50%)
3	1 (13%)
4	1 (13%)
Metastatic sites other than brain, *N* (%)	
Lung	3 (38%)
Bone	2 (25%)
Liver	1 (13%)
Others	4 (50%)
Systemic disease not under control, *N* (%)	5 (63%)

### Treatment administration

The mean number of cycles administered was 3.3 (median, 3.0). Only 2 of 8 patients (25%) completed the planned 6 cycles of treatments. The reasons that 6 patients did not complete the study are listed as follows: one patient (13%) exhibited both CNS and extra-CNS disease progression; one patient (13%) exhibited only extra-CNS disease progression; one patient (13%) exhibited no CSF cytologic response; and 3 patients (38%) withdrew from the study. Six patients concurrently received intrathecal methotrexate therapy. The treatment course of each patient is shown in the Additional file 1.

### Safety

The AEs are listed in Table 2. The most common grade 3/4 AEs were neutropenia (23.1%), leukopenia (23.1%), and hyponatremia (23.1%). Nonhematologic toxicity was generally modest. All AEs resolved to grade 1 or lower.

**Table 2:** **Numbers of major adverse events of the indicated grade (total 26 cycles)**

	Grade 1/2	Grade 3/4	All grades			
	*N*	%	*N*	%	*N*	%
Hematological						
Neutrophil count decreased	9	34.6	6	23.1	15	57.7
Lymphocyte count decreased	9	34.6	6	23.1	15	57.7
Anemia	13	50.0	0	0.0	13	50.0
Platelet count decreased	10	38.5	1	3.8	11	42.3
Non-hematological						
Hypertension	14	53.8	0	0.0	14	53.8
ALT/AST increased	6	23.1	2	7.7	8	30.8
Headache	8	30.8	0	0.0	8	30.8
Hyponatremia	1	3.8	6	23.1	7	26.9
Proteinuria	7	26.9	0	0.0	7	26.9

### Efficacy

The patient characteristics and their outcomes are listed in the Table 3. Because 3 patients withdrew from the study before undergoing follow-up CSF studies and neurologic assessments, the CNS-specific response was evaluable in 5 patients. Three patients (60%) were responsive, exhibiting the clearance of malignant cells in successive CSF studies, and 2 of them completed the planned treatment courses. One patient was considered nonresponsive because positive cytology results were observed after a single negative cytology result was obtained, and one patient’s CSF cytology results were persistently positive. Clinically, 3 patients (60%) improved neurologically without evidence of systemic progression; one patient (20%) was neurologically stable but progressed systemically; and one patient (20%) exhibited both neurologic and systemic progression.

**Table 3:** **Clinical characteristics and outcomes of the patients**

Case	Age	Histology	CSF cytology response	Neurological assessment	CNS response	Extra-CNS response	PFS (m)	OS (m)
1	53	ILC	Yes	Improved	Responder	No PD	10.7	10.7
2	65	NA	NA	NA	NA	NA	0.7	0.7*
3	35	IDC	No	Improved	Non-responder	No PD	7.6	7.6
4	63	IDC	Yes	Improved	Responder	No PD	9.0	9.0
5	63	IDC	Yes	Improved	Responder	Liver PD	4.7	4.7
6	30	IDC	No	Progressed	Progression	Breast PD	2.9	2.9
7	49	IDC	NA	NA	NA	NA	0.7	0.7
8	56	IDC	NA	NA	NA	NA	1.6	1.6

*Censored.

*Abbreviations: ILC* invasive lobular carcinoma, *NA* not available, *IDC* invasive ductal carcinoma, *PD* progressive disease, *Bev* bevacizumab administration, *PFS* progression free survival, *OS* overall survival.

**Figure 1:**
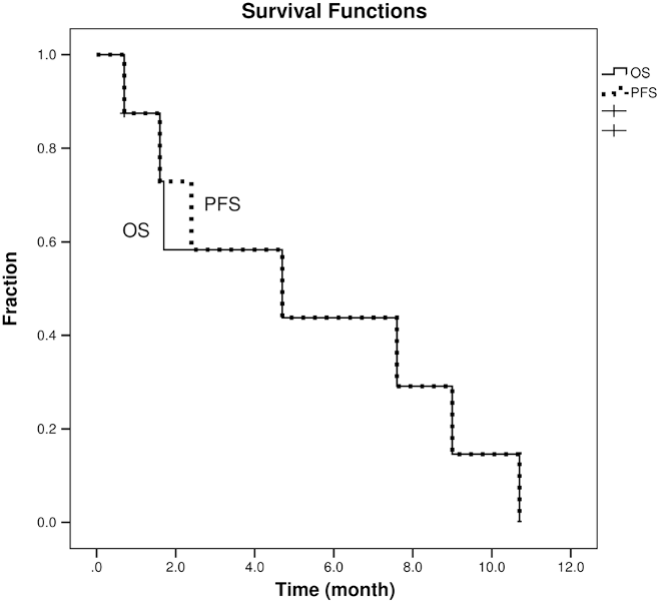
Efficacy results: Overall Survival (OS) and neurologic progression free survival (PFS).

The 8 patients were subjected to a survival analysis according to the intent-to-treat analysis. The median OS was 4.7 months (95% CI 0.3–9.0; Figure 1). The responders of CSF cytology had a trend toward longer median overall survival (9.0 vs 2.9 months, *P =* 0.076). The OS of the 3 responders was 10.7, 9.0, and 4.7 months respectively. The neurologic PFS was 4.7 months (95% CI 0–10.5; Figure 1).

### Cerebrospinal fluid concentration of etoposide

**Figure 2:**
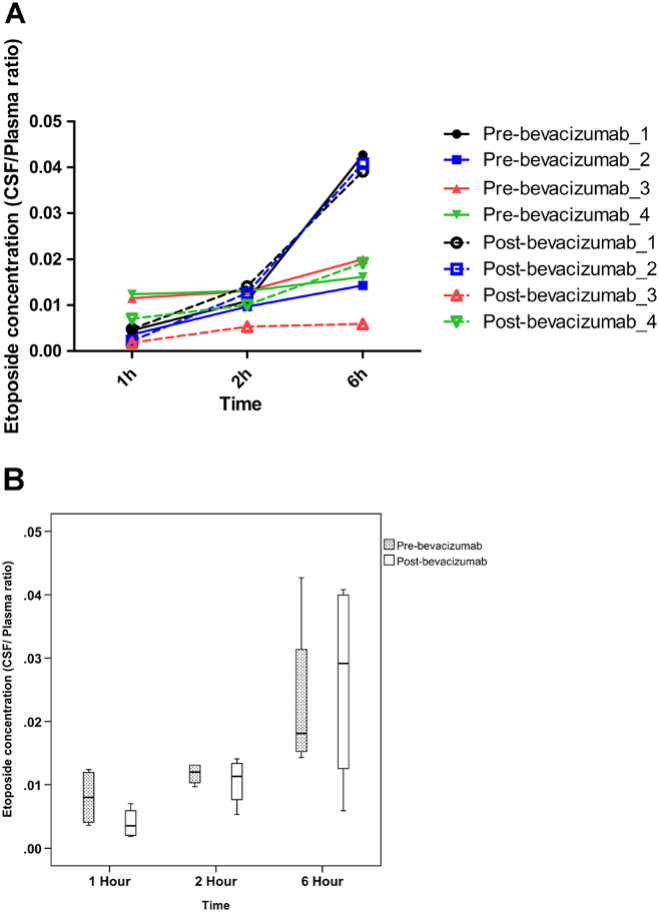
The effects of bevacizumab administration on the temporal changes of CSF to plasma ratio of etoposide concentration. **(A)** Individual CSF/ Plasma ratio of etoposide concentration versus time plot. **(B)** Overall CSF/Plasma ratio of etoposide concentration versus time plot.

Four patients were subjected to serial measurements of etoposide concentrations in the CSF and plasma before and after bevacizumab administration. A plot of the individual ratio of the etoposide concentration in the CSF to that in the plasma versus time is shown in Figure 2A. We observed that bevacizumab administered 24 hours prior to the administration of cytotoxic drugs exerted no significant effects on etoposide penetration into the CSF (Figure 2B, *P* = .167, .680, .754, at 1 h, 2 h, and 6 h, respectively).

## Discussion

Several retrospective studies and case reports have demonstrated the feasibility of using systemic therapies, including capecitabine, lapatinib, gefitinib, erlotinib, and bevacizumab therapies, in treating leptomeningeal carcinomatosis [12-17]. However, only a few prospective clinical trials have been conducted [18-22] (Table 4). This is partly because of the difficulties in conducting a large trial among patients with extremely poor prognoses, for whom treatment may be discontinued early, precluding a full assessment of the agents that exhibit potential activity in treating this disease.

**Table 4:** **Prospective clinical trials of systemic therapy for leptomeningeal carcinomatosis**

Drugs	Study	Primary tumor	Phase	*N*	Efficacy
Methotrexate	[19]	Breast, lung	I	13	0% cytologic and clinical response.
Temozolomide	[22]	Breast, lung, melanoma	II	19	11% cytologic or radiological response; TTP 28 days.
Topotecan and ifosfamide	[20]	Breast, lung	II	7	28% radiological response; TTP 51 days; OS 218 days.
Patupilone	[21]	Breast	II	5	0% cytologic and radiologic response; 3 months CNS PFS rate, 20%.
Bevacizumab	[23]	Breast, lung, melanoma	II	15 (Ongoing)	7% best protocol responses; 13% CSF response; PFS 6 weeks; mOS 14 weeks.
Bevacizumab, etoposide and cisplatin	Wu *et al.*, 2015	Breast	II	8	60% CNS-specific response rate; OS 4.7 mos.

According to our thorough review of research, this is the first prospective pilot study to report on the efficacy of anti-VEGF therapy plus chemotherapy in leptomeningeal carcinomatosis. Specifically, the CNS-specific response rate was 60% and the median OS was 4.7 months (95% CI 0.3–9.0). Groves *et al.* [23] reported that administration of bevacizumab alone yielded a 13% CSF response and a median OS of 14 weeks. Because of the heterogeneity of the enrolled patients and differences in response criteria among studies, comparing the efficacy of various systemic treatments is difficult (Table 4). In addition, the ability to make statistically sound conclusions was limited by the small sample size of our study. Because this patient population is seldom included in clinical trials, any treatment with evidence of response warrants further investigation.

Among the 8 patients enrolled in this study, 3 patients dropped out during the early phase of the trial due to patients’ refusal of continued treatments. Only 5 patients underwent follow-up CSF studies and neurologic assessments, and were evaluable for the CNS-specific response. In addition to excluding the 3 dropouts in the final analysis, the response rates could also be estimated by assuming that the 3 dropped patients were non-responders, that is, 3/8 (38%). In this way, it underestimated the true response rate and could be considered as a low bound of the estimated response rate based on the data of this study. Similarly, the PFS could also be analyzed in two ways by including (1) all 8 patients, and (2) only the 5 patients who completed the response evaluation. The former included the 3 dropped patients who did not complete the response evaluation, and thus it also underestimated the effect of the proposed treatment on PFS (4.7 months versus 7.6 months) and could be considered as a low bound of the estimated effect of the proposed treatment on PFS based on the data of this study.

Intrathecal methotrexate has been used for a long time, but its value is questioned, with median survival of about 7–16 weeks in previous reports (7 weeks, Fizazi *et al*.; 11 weeks, Glantz *et al*.; 16 weeks, Rudnicka *et al.* and Grossman *et al.*) [11,24-26]. The median overall survival in our study was 4.7 months, which seems slightly better than intrathecal methotrexate treatment in previous serials. Although the efficacy of bevacizumab-based therapy might be confounded by the concurrent intrathecal methotrexate therapy administered in this study, the observation that one patient was responsive to BEEP rechallenge while disease progressed under maintenance intrathecal methotrexate therapy provides evidence that BEEP can benefit leptomeningeal carcinomatosis patients (Table 3, Patient 1).

Increasing evidence suggests that abnormal tumor vasculature can hinder effective cancer therapy; furthermore, VEGF inhibition can transiently normalize tumor vasculature and improve tumor perfusion as well as the delivery of subsequent chemotherapy [27-29]. In the study conducted by Dickson *et al.*, the penetration of chemotherapy was improved when it was administered 1 to 3 days after bevacizumab administration in the neuroblastoma xenograft model [29]. Although Van der Veldt *et al.* observed that bevacizumab reduced perfusion and the uptake of trace amounts of [^11^C] docetaxel in NSCLC tissues within 5 hours to at least 4 days, the effects of bevacizumab on microdoses of drug delivery in tumors may not hold true for pharmacological drug concentrations [30].

In the present study, cytotoxic drugs were administered 24 hours after the administration of bevacizumab to enhance efficacy based on the normalization theory [29-31]. We observed that anti-VEGF therapy exerted no significant effects on the penetration of etoposide into the CSF. Additional studies are required to clarify whether different schedules for treatments in which bevacizumab is combined with cytotoxic agents increase drug penetration into the CSF and improve the treatment efficacy.

## Conclusions

BEEP regimen exhibited promising efficacy in breast cancer patients with leptomeningeal carcinomatosis. Additional studies are warranted to verify the efficacy of the regimen and clarify the role of bevacizumab in this disease.

## Additional file

Additional file 1:
**The treatment course of each patient.**

## Abbreviations

VEGF: Elevated vascular endothelial growth factor
BEEP: Bevacizumab combined with etoposide and cisplatin
CNS: Central nervous system
CSF: Cerebrospinal fluid
95% CI: 95% confidence interval
OS: Overall survival
NSCLC: Non-small cell lung cancer
G-CSF: Granulocyte colony-stimulating factor
AE: Adverse events
PFS: Progression-free survival
