# Supplementary figures and images for: A pilot study of bevacizumab combined with etoposide and cisplatin in breast cancer patients with leptomeningeal carcinomatosis
Source: BMC Cancer. 2015 Apr 17;15:299. doi: 10.1186/s12885-015-1290-1 (PMC4403836; doi:10.1186/s12885-015-1290-1)

**Additional file 1**

S1: The treatment course of each patient.

: IT Methotrexate; : BEEP.


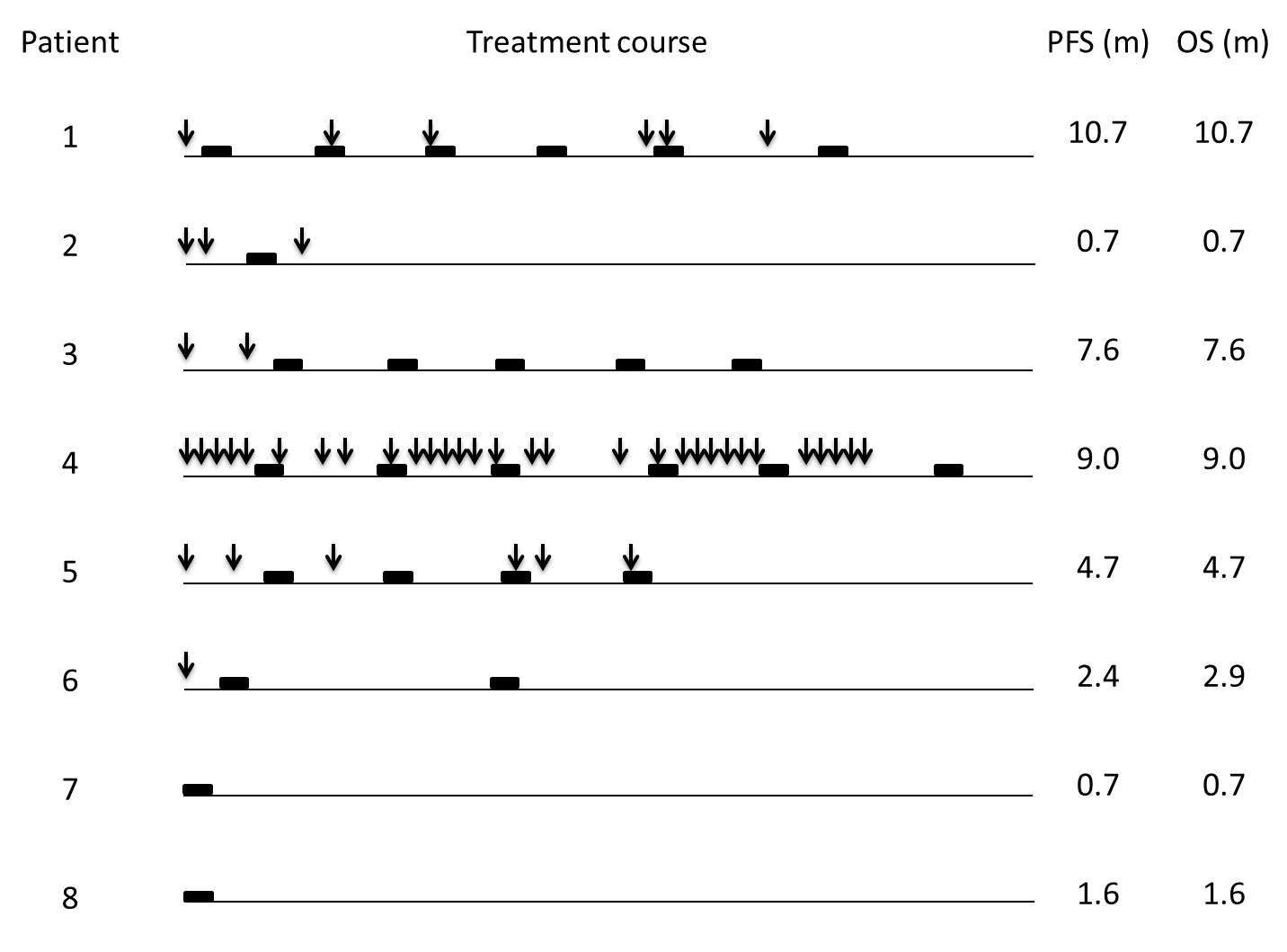

Supplement: Additional file 1: — The treatment course of each patient. [file 12885_2015_1290_MOESM1_ESM.docx]
